# Standardized Saffron Extract Formulation for Low Mood and Subclinical Depressive Symptoms: A Randomized, Double-Blind, Placebo-Controlled Trial

**DOI:** 10.7759/cureus.110935

**Published:** 2026-06-15

**Authors:** Shishir Shah, Nishesh Shah, Jai Shah, Dyuthi S, Janani S, P Sudha Rani

**Affiliations:** 1 Department of Product Development, Xtractiva Life Science Private Limited, Kochi, IND; 2 Department of Research and Development, Universal Oleoresins, Kochi, IND; 3 Department of Psychiatry, Meenakshi Multispeciality Hospital, Chennai, IND; 4 Department of Clinical Research, Meenakshi Multispeciality Hospital, Chennai, IND

**Keywords:** crocus sativus, depressive symptoms, low mood, mood regulation, saffron, serum cortisol, sleep quality, subclinical depression

## Abstract

Background and aim

Low mood with subclinical to mild depressive symptoms represents an early stage of mood disorders with risk of progression to major depression. This study was conducted to evaluate the efficacy and safety of a standardized saffron extract formulation (UO SAF 02, 15 mg) in adults with self-reported low mood and subclinical to mild depressive symptoms.

Methods

In this randomized, double-blind, placebo-controlled trial, 56 adults aged 18-65 years with self-reported low mood and Beck Depression Inventory (BDI) scores of 10-20 were randomized (1:1) to receive UO SAF 02 or placebo for 56 days. The trial was registered with the Clinical Trials Registry of India (CTRI/2023/11/059603).

The primary outcome was the change in total Profile of Mood States (POMS) scores. Secondary outcomes included measures of affective state (Positive and Negative Affect Schedule (PANAS)), depression, anxiety, and stress (Depression Anxiety Stress Scales (DASS-21)), sleep quality (Pittsburgh Sleep Quality Index (PSQI)), and serum cortisol levels.

Results

Compared with placebo, UO SAF 02 demonstrated significant and consistent improvements in mood-related outcomes, with statistically significant effects observed at Day 28 and maintained through Day 56. POMS scores decreased markedly in the UO SAF 02 group at Day 56 (-48.46 ± 5.39, p < 0.0005) versus the placebo group (-5.67 ± 3.44, p > 0.05). Significant improvements were also observed in affective states, with greater increases in positive affect and reductions in negative affect (p < 0.05), along with reductions in DASS-21 scores (p < 0.001) and improvements in sleep quality (PSQI; p < 0.05). Serum cortisol levels were significantly reduced at both Day 28 and Day 56 (p < 0.05). The intervention was well tolerated, with no serious adverse events reported.

Conclusions

UO SAF 02 significantly improved psychological and physiological mood-related outcomes in individuals with low mood and subclinical to mild depressive symptoms, supporting its potential as an effective and well-tolerated intervention for mood regulation.

## Introduction

Low mood is a commonly reported subjective experience in the general population and is often characterized by transient feelings of sadness, reduced motivation, or diminished emotional well-being [1]. A considerable proportion of individuals experiencing low mood also exhibit measurable depressive symptoms that fall within the subclinical to mild range, but do not meet diagnostic criteria for major depressive disorder (MDD) [1]. Such subclinical depressive symptoms are characterized by the presence of depressive features with lower symptom severity, fewer symptoms, and limited functional impairment, falling below the threshold required for a formal clinical diagnosis [2,3]. In contrast, MDD is typically diagnosed when an individual experiences five or more depressive symptoms, including depressed mood and/or loss of interest or pleasure, for at least two weeks and with clinically significant distress or functional impairment [4].

The subclinical presentations are increasingly recognized as clinically meaningful, as they are associated with impaired quality of life and a higher risk of progression to clinically diagnosed depression [5-7]. Low mood with subclinical depressive symptoms may therefore represent an early stage along the continuum of mood disorders, emphasizing the importance of timely identification and management to prevent further progression [7,8].

Despite its clinical relevance and high prevalence, effective treatment options for individuals with low mood and subclinical depressive symptoms remain limited. Conventional antidepressants are primarily indicated for moderate to severe depression and are associated with delayed onset of action, potential adverse effects, and adherence challenges [9-11]. Furthermore, pharmacological treatment may not be considered appropriate for individuals with milder symptoms, resulting in a therapeutic gap for this population [12,13]. These limitations have led to increasing interest in identifying safe, well-tolerated, and accessible interventions that can support mood regulation and potentially prevent progression to more severe depressive disorders [9].

Saffron (*Crocus sativus* L.) has emerged as a promising natural intervention for mood disorders. It is a spice derived from the dried stigmas of the saffron crocus and contains several bioactive constituents, of which crocin, crocetin, picrocrocin, and safranal are considered the principal compounds responsible for its pharmacological effects [14,15]. A growing body of evidence from randomized controlled trials and meta-analyses indicates that saffron supplementation significantly reduces depressive symptoms, with efficacy comparable to conventional antidepressants in individuals with mild to moderate depression [16-18]. Meta-analytic findings further demonstrate large effect sizes of saffron compared with placebo in improving depressive symptoms [19]. In addition to its antidepressant effects, saffron has also shown benefits in related domains, including anxiety, stress, sleep quality, and overall emotional well-being, across both clinical and non-clinical populations [20-23]. It also exhibits a favorable safety profile and broad neuropsychotropic effects, supporting its role as a well-tolerated intervention for mood-related conditions [24].

Despite the expanding evidence base, most clinical studies on saffron have focused on individuals with clinically diagnosed depression. Evidence evaluating its efficacy in individuals with self-reported low mood and subclinical to mild depressive symptoms remains limited and less well characterized [22,25]. Given that this subset represents a large and often underserved population at risk of developing more severe mood disorders, there is a clear need for well-designed clinical trials evaluating safe and effective interventions tailored to this group.

The investigational product evaluated in the present study, UO SAF 02 (15 mg), is a saffron extract formulation standardized to key bioactive constituents, including crocin (not less than 11%; equivalent to ≥1.65 mg per capsule) and safranal (not less than 0.1%; equivalent to ≥0.015 mg per capsule). This formulation is developed using a proprietary solid-liquid fusion technology (SOLIFUZE®, Xtractiva Lifescience Pvt. Ltd, Kochi, Kerala, India), designed to enhance the stability and bioavailability. UO SAF 02 is the internal formulation code assigned to the investigational standardized saffron extract product. The present study aimed to evaluate the efficacy and safety of UO SAF 02 in adults with self-reported low mood and subclinical-to-mild depressive symptoms, with primary assessment focused on overall mood disturbance and secondary evaluation of affective state, stress, sleep quality, and a physiological stress marker.

## Materials and methods

Study design and ethical considerations

This was a randomized, double-blind, placebo-controlled, parallel-group clinical trial conducted at Meenakshi Multispeciality Hospital, Mylapore, Chennai, India, between November 2023 and February 2024. The study was designed to evaluate the efficacy and safety of a novel saffron extract formulation in adults with self-reported low mood and subclinical to mild depressive symptoms.

The study protocol was reviewed and approved by the ethics committee of Meenakshi Multispeciality Hospital, Chennai (Ref No: CMMHEC/23/06; dated September 25, 2023) prior to initiation. The study was conducted in accordance with the ethical principles outlined in the Declaration of Helsinki, the International Council for Harmonization Good Clinical Practice (ICH-GCP) guidelines, and applicable national regulatory requirements. The study was prospectively registered with the Clinical Trials Registry of India (CTRI/2023/11/059603; dated November 7, 2023).

Written informed consent was obtained from all participants before enrollment, and participant confidentiality was maintained throughout the study.

This randomized controlled trial was conducted and reported in accordance with the Consolidated Standards of Reporting Trials (CONSORT) guidelines.

Study participants

Participants were recruited from outpatient clinical settings through voluntary participation and screened for eligibility using a convenience sampling approach. Eligible participants were healthy adult males and females aged 18-65 years, with a body mass index (BMI) <30 kg/m², who self-reported low mood during initial screening. Of these individuals, those with the Beck Depression Inventory (BDI) scores ranging from 10 to 20, indicative of subclinical to mild depressive symptoms, were included in the study. BDI is a 21-item self-reported instrument comprising cognitive, affective, and somatic items, with total scores ranging from 0 to 63, where higher scores indicate greater severity of depressive symptoms; it is a widely validated and reliable measure of depressive symptomatology [26]. The BDI was used for screening purposes and was not included as a longitudinal outcome measure. Participants did not have a formal clinical diagnosis of depressive disorder.

Participants were excluded if they had a diagnosis of depressive or other mood disorders (e.g., major depressive disorder or bipolar disorder), were receiving medications affecting mood, cognition, or neuroendocrine function, or had significant hepatic, renal, cardiovascular, or neurological disorders. Additional exclusions included substance or alcohol abuse, hypersensitivity to herbal or nutritional products, anticoagulant use, pregnancy or lactation, participation in another clinical trial within 30 days, or any condition that could interfere with study participation or outcome assessment.

Investigational product

The investigational product was a novel saffron extract formulation (UO SAF 02, 15 mg), developed using a proprietary solid-liquid fusion technology designed to enhance the stability and bioavailability of key bioactive constituents. The formulation was standardized to contain crocin (not less than 11%; equivalent to ≥1.65 mg per capsule) and safranal (not less than 0.1%; equivalent to ≥0.015 mg per capsule), quantified using UV spectroscopy and high-performance liquid chromatography (HPLC), respectively. This delivery approach supports enhanced bioavailability and sustained release of active components, potentially improving the overall efficacy of the formulation without reliance on excessively high concentrations of bioactives.

Randomization

Participants were randomized in a 1:1 ratio to receive either the investigational product or a matching placebo. Randomization was performed using a computer-generated block randomization schedule prepared by an independent statistician to ensure balanced allocation between treatment groups. Allocation concealment was ensured using sequentially numbered, sealed, opaque envelopes containing the group assignments, which were maintained at the study site and opened sequentially only after participant enrollment. The study personnel responsible for participant enrollment did not have prior access to the allocation sequence. Both participants and investigators were blinded to treatment allocation. The investigational product and placebo were identical in appearance, packaging, and labeling to ensure blinding. Participants were instructed to take the assigned intervention orally as capsules at a dose of 15 mg twice daily (morning and evening) with food for 56 days. The study intervention was self-administered by participants at their residences. The dosing regimen was consistent across all participants within the eligible age range (18-65 years), with no age-based dose modifications.

Study procedures and assessments

The study consisted of four visits: screening (Day -7 to Day 0), baseline/randomization (Day 1), interim assessment (Day 28), and end-of-study assessment (Day 56). At screening, participants were evaluated for eligibility based on the predefined inclusion and exclusion criteria. At baseline, demographic and clinical data were collected, and participants were randomized and initiated on study treatment. Follow-up visits at Days 28 and 56 included assessments of depressive symptoms, mood-related outcomes, and safety parameters. Compliance was monitored through participant diaries and counts of returned study products. Validated psychometric instruments were used to assess depressive symptoms and mood-related outcomes, while sleep quality and stress-related parameters were also evaluated. Safety assessments included monitoring of vital signs (blood pressure, pulse rate), laboratory parameters (hematology, biochemical parameters, and urine analysis), physical/systemic examinations, adverse events, and concomitant medication use at scheduled study visits throughout the study period. Clinically significant changes were defined as values outside the normal reference range, changes requiring medical intervention, or findings associated with clinical symptoms.

Outcome measures

The primary efficacy outcome was the change in mood-related outcomes from baseline to Day 56, as assessed using the Profile of Mood States (POMS), a validated self-reported instrument comprising multiple domains of mood disturbance (including tension, depression, anger, vigor, fatigue, and confusion), with higher total scores indicating greater mood disturbance [27]. Secondary outcomes included changes in affective state, depression, anxiety, stress, sleep quality, and serum cortisol levels. Affective state was assessed using the Positive and Negative Affect Schedule (PANAS) [28], a validated 20-item self-reported instrument comprising positive affect and negative affect subscales, with higher scores indicating greater levels of the respective affective states. Depression, anxiety, and stress were assessed using the Depression Anxiety Stress Scales (DASS-21) [29], a validated 21-item self-reported instrument comprising depression, anxiety, and stress subscales, with higher scores indicating greater symptom severity. Sleep quality was assessed using the Pittsburgh Sleep Quality Index (PSQI) [30], a validated self-reported instrument evaluating multiple domains of sleep quality, with higher global scores indicating poorer sleep quality. Serum cortisol levels were measured as a physiological marker of stress using standard laboratory methods under fasting morning conditions, and values were expressed in µg/dL. Both primary and secondary outcome measures were assessed at baseline (Day 1), interim (Day 28), and end-of-study (Day 56).

Safety analyses were conducted on all participants who received at least one dose of the study intervention. Adverse events (AEs), adverse drug reactions (ADRs), and serious adverse events (SAEs) were summarized by system organ class and preferred term using the Medical Dictionary for Regulatory Activities (MedDRA) [31]. Events leading to discontinuation were also recorded.

Sample size calculation

The sample size was calculated to detect a clinically meaningful difference in the primary outcome between the treatment groups, with 80% power and a two-sided significance level of 0.05. The estimation was based on anticipated effect sizes derived from prior evidence in similar study settings. Based on these assumptions, a total sample size of 56 participants was considered adequate for the study, with 28 participants allocated to each group.

Statistical analysis

The primary efficacy analysis was conducted on the intention-to-treat (ITT) population, defined as all participants who received at least one dose of the study intervention and had at least one post-baseline assessment. The per-protocol (PP) population was defined but not analyzed separately, as its results were consistent with those of the ITT population. Missing data were handled using the last observation carried forward (LOCF) method, given the minimal extent of missing data and the study sample size. Given the minimal missing data (n=3; 5.4%) and balanced distribution across groups, LOCF was considered unlikely to materially influence study outcomes.

Primary and secondary outcomes were analyzed using repeated measures analysis of variance (ANOVA) to assess changes over time within groups, and independent sample t-tests were used for between-group comparisons at each time point. Changes from baseline were calculated as the difference between post-treatment and baseline values. Effect sizes were calculated using change scores and pooled standard deviations; interpretation should consider the relatively low baseline variability and the homogeneous study population, which may result in larger standardized effect estimates.

All statistical analyses were performed using IBM SPSS Statistics for Windows, version 28 (IBM Corp., Armonk, NY, USA). Continuous variables are presented as mean ± standard deviation (SD), and categorical variables as frequencies and percentages. A two-sided p-value of <0.05 was considered statistically significant.

## Results

Participant disposition

**Figure 1:**
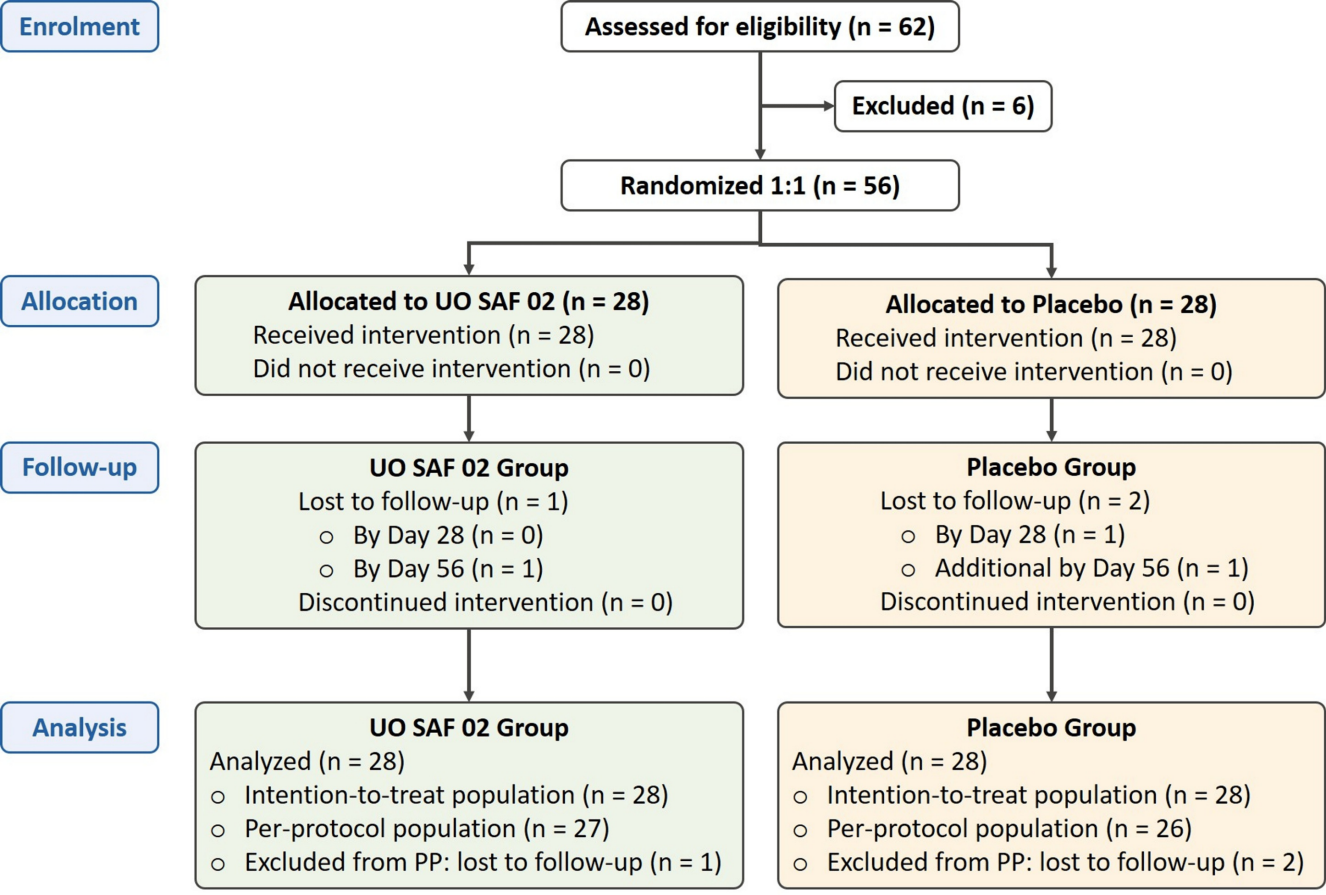
Participant disposition (CONSORT flow diagram) PP: per-protocol population; CONSORT: Consolidated Standards of Reporting Trials

A total of 62 participants were screened, of whom 56 met eligibility criteria and were randomized in a 1:1 ratio to receive UO SAF 02 or placebo (n = 28 per group). Fifty-three participants (94.6%) completed the study, including 27 in the UO SAF 02 group and 26 in the placebo group (Figure 1).

A total of three participants were lost to follow-up. In the UO SAF 02 group, one participant discontinued after the interim assessment (Day 28) and did not complete the final visit (Day 56). In the placebo group, one participant did not attend the interim assessment (Day 28), while another discontinued after the interim assessment prior to the final visit. Missing outcome data resulting from these losses were handled using the LOCF method.

No discontinuations due to adverse events were reported. Overall treatment compliance exceeded 85% in both groups, indicating good adherence to the study intervention.

Baseline characteristics

Baseline demographics were comparable between groups, indicating successful randomization. No statistically significant differences were observed in age, gender distribution, body mass index (BMI), or baseline efficacy measures (Table 1).

**Table 1:** Baseline demographic (ITT population) Data are presented as mean ± SD or number (%). Between-group comparisons were performed using independent sample t-tests for continuous variables and chi-square tests for categorical variables. POMS: Profile of Mood States; PANAS: Positive and Negative Affect Schedule; DASS-21: 21-item Depression Anxiety Stress Scales; PSQI: Pittsburgh Sleep Quality Index; ITT: intention to treat

Parameter	UO SAF 02 (n = 28)	Placebo (n = 28)	p-value
Age (years), mean ± SD	28.54 ± 8.88	30.04 ± 9.13	> 0.05
Female, n (%)	18 (64.3%)	24 (85.7%)	> 0.05
Male, n (%)	10 (35.7%)	4 (14.3%)	> 0.05
Body weight (kg), mean ± SD	59.41 ± 10.01	59.24 ± 9.77	> 0.05
Height (cm), mean ± SD	161.23 ± 7.12	159.79 ± 7.12	> 0.05
Body mass index (kg/m²), mean ± SD	22.76 ± 2.94	23.11 ± 2.86	> 0.05
POMS total score	69.54 ± 1.50	69.44 ± 1.25	> 0.05
PANAS Positive Affect	20.14 ± 1.27	20.37 ± 1.24	> 0.05
PANAS Negative Affect	25.32 ± 0.94	25.33 ± 0.96	> 0.05
DASS-21 total score	23.79 ± 1.17	22.37 ± 2.15	> 0.05
PSQI score	14.68 ± 0.77	14.63 ± 0.74	> 0.05

Primary outcome

Profile of Mood States (POMS)

A progressive, statistically significant reduction in POMS scores was observed in the UO SAF 02 group compared with the placebo group over the study period (Table 2), indicating a substantial improvement in mood-related symptoms.

**Table 2:** Changes in Profile of Mood States (POMS) scores from baseline to Day 56 (ITT population) Data presented as Mean ± SD. Δ indicates change from baseline. Between-group comparisons were performed using independent sample t-tests. 95% confidence intervals and Cohen's d values were calculated for between-group differences in change scores. ITT: intention to treat

Timepoint	UO SAF 02 (Mean ± SD) (n = 28)	Placebo (Mean ± SD) (n = 28)	Mean difference (95% CI)	Between-group p-value	Effect size (Cohen's d)
Baseline	69.54 ± 1.50	69.44 ± 1.25	—	—	—
Day 28	40.32 ± 2.80	65.70 ± 2.02	—	<0.001	—
Day 56	21.07 ± 5.81	63.78 ± 3.49	—	<0.001	—
Δ Day 28	-29.21 ± 2.78	-3.74 ± 1.87	-25.47 (-26.72 to -24.20)	<0.001	10.75
Δ Day 56	-48.46 ± 5.39	-5.67 ± 3.44	-42.79 (-45.20 to -40.38)	<0.001	9.47

Within-group analysis demonstrated significant reductions from baseline at both Day 28 and Day 56 in the UO SAF 02 group (p < 0.001), reflecting an early onset of effect that was further sustained over time. In contrast, the placebo group showed only minimal, non-significant changes over the study duration (p > 0.05). The consistent downward trajectory of POMS scores in the UO SAF 02 group suggests clinically meaningful improvement in the primary outcomes.

Between-group differences were statistically significant on both Day 28 and Day 56 (p < 0.05), with a substantially greater magnitude of improvement in the UO SAF 02 group. The between-group differences in change from baseline were large, with mean differences of -25.47 (95% CI: -26.72 to -24.20; Cohen’s d = 10.75) at Day 28 and -42.79 (95% CI: -45.20 to -40.38; Cohen’s d = 9.47) at Day 56, supporting a robust treatment effect (Table 2).

Secondary outcomes

Positive and Negative Affect (PANAS)

Changes in affective states followed a clear time-dependent pattern. Treatment with UO SAF 02 resulted in significant and sustained improvements in both positive and negative affect (Table 3).

**Table 3:** Changes in Positive and Negative Affect Schedule (PANAS) scores from baseline to Day 56 (ITT population) Data presented as Mean ± SD. Δ indicates change from baseline. Between-group comparisons were performed using independent sample t-tests. 95% confidence intervals and Cohen's d values were calculated for between-group differences in change scores. ITT: intention to treat

Timepoint	UO SAF 02 (Mean ± SD) (n = 28)	Placebo (Mean ± SD) (n = 28)	Mean difference (95% CI)	Between-group p-value	Effect size (Cohen's d)
Positive Affect scores					
Baseline	20.14 ± 1.27	20.37 ± 1.24	—	—	—
Day 28	25.25 ± 1.35	22.07 ± 2.16	—	<0.001	—
Day 56	31.11 ± 1.81	22.56 ± 2.24	—	<0.001	—
Δ Day 28	+5.11 ± 1.32	+1.70 ± 1.85	3.41 (2.44 to 4.38)	<0.001	2.14
Δ Day 56	+10.96 ± 2.08	+2.19 ± 2.32	8.77 (7.58 to 9.96)	<0.001	3.98
Negative Affect scores					
Baseline	25.32 ± 0.94	25.33 ± 0.96	—	—	—
Day 28	18.11 ± 1.17	23.33 ± 2.04	—	<0.001	—
Day 56	10.32 ± 2.39	22.19 ± 2.09	—	<0.001	—
Δ Day 28	-7.21 ± 1.10	-2.00 ± 1.80	-5.21 (-6.12 to -4.30)	<0.001	3.38
Δ Day 56	-15.00 ± 2.43	-3.15 ± 2.13	-11.85 (-13.05 to -10.65)	<0.001	5.18

Within-group analysis showed a statistically significant increase in positive affect and a reduction in negative affect at both Day 28 and Day 56 in the UO SAF 02 group (p < 0.001), indicating both early onset and progressive improvement in affective states. In contrast, changes observed in the placebo group were minimal and not statistically significant.

Between-group comparisons confirmed significant differences at both post-baseline time points (p < 0.001), with improvements favoring UO SAF 02. The magnitude of effect was substantial at Day 56, with positive affect showing a between-group difference of 8.77 (95% CI: 7.58 to 9.96; Cohen’s d = 3.98) and negative affect showing a reduction of -11.85 (95% CI: -13.05 to -10.65; Cohen’s d = 5.18), indicating a robust treatment effect (Table 3).

Depression, Anxiety, and Stress (DASS-21) Total Score

**Figure 2:**
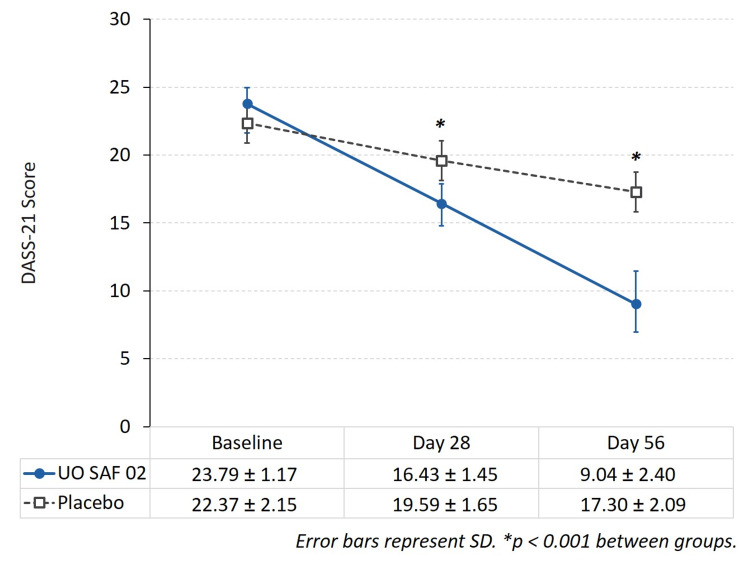
Changes in Depression, Anxiety, and Stress Scale-21 (DASS-21) total scores from baseline to Day 56 (ITT population) ITT: intention to treat

Total DASS-21 scores were analyzed as a composite measure of overall psychological distress. The total DASS-21 scores demonstrated a clear and progressive reduction in the UO SAF 02 group over the course of the study, indicating significant improvement across depression, anxiety, and stress domains (Table 4, Figure 2).

**Table 4:** Changes in Depression, Anxiety, and Stress Scale-21 (DASS-21) total scores from baseline to Day 56 (ITT population) Data presented as Mean ± SD. Δ indicates change from baseline. Between-group comparisons were performed using independent sample t-tests. 95% confidence intervals and Cohen's d values were calculated for between-group differences in change scores. ITT: intention to treat

Timepoint	UO SAF 02 (Mean ± SD) (n = 28)	Placebo (Mean ± SD) (n = 28)	Mean difference (95% CI)	p-value	Effect size (Cohen's d)
Baseline	23.79 ± 1.17	22.37 ± 2.15	—	—	—
Day 28	16.43 ± 1.45	19.59 ± 1.65	—	< 0.001	—
Day 56	9.04 ± 2.40	17.30 ± 2.09	—	< 0.001	—
Δ Day 28	-7.36 ± 1.77	-2.78 ± 2.06	-4.58 (-5.60 to -3.56)	< 0.001	2.39
Δ Day 56	-14.75 ± 2.47	-5.07 ± 2.72	-9.68 (-11.12 to -8.24)	< 0.001	3.72

Within-group analysis demonstrated statistically significant reductions from baseline at both Day 28 and Day 56 in the UO SAF 02 group (p < 0.001), whereas the placebo group exhibited only modest, non-significant changes. These findings indicate an early therapeutic effect that was sustained over time.

Between-group comparisons also revealed statistically significant differences at both post-baseline time points (p < 0.001), with a substantially greater improvement observed in the UO SAF 02 group. Notably, these differences were evident by Day 28 and became more pronounced at Day 56. At Day 56, the between-group difference in change from baseline was -9.68 (95% CI: -11.12 to -8.24; Cohen’s d = 3.72), indicating a substantial treatment effect (Table 4).

Sleep Quality (PSQI)

**Figure 3:**
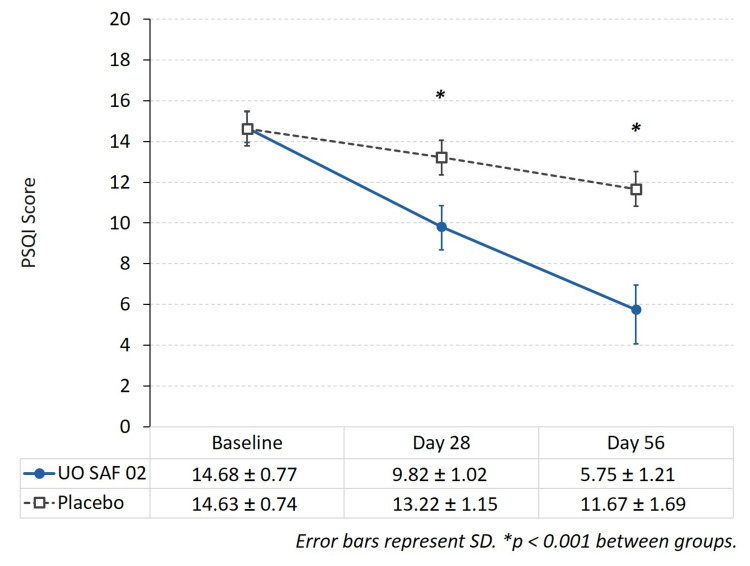
Changes in Pittsburgh Sleep Quality Index (PSQI) scores from baseline to Day 56 (ITT population) ITT: intention to treat

Treatment with UO SAF 02 resulted in significant and sustained improvements in sleep quality, as evidenced by reductions in PSQI scores over time (Table 5, Figure 3).

**Table 5:** Changes in Pittsburgh Sleep Quality Index (PSQI) scores from baseline to Day 56 (ITT population) Data presented as Mean ± SD. Δ indicates change from baseline. Between-group comparisons were performed using independent sample t-tests. 95% confidence intervals and Cohen's d values were calculated for between-group differences in change scores. ITT: intention to treat

Timepoint	UO SAF 02 (Mean ± SD) (n = 28)	Placebo (Mean ± SD) (n = 28)	Mean difference (95% CI)	p-value	Effect size (Cohen's d)
Baseline	14.68 ± 0.77	14.63 ± 0.74	—	—	—
Day 28	9.82 ± 1.02	13.22 ± 1.15	—	< 0.001	—
Day 56	5.75 ± 1.21	11.67 ± 1.69	—	< 0.001	—
Δ Day 28	-4.86 ± 0.80	-1.41 ± 0.69	-3.45 (-3.82 to -3.08)	< 0.001	4.58
Δ Day 56	-8.93 ± 1.18	-2.96 ± 1.37	-5.97 (-6.64 to -5.30)	< 0.001	4.66

Within-group analysis showed statistically significant reductions from baseline at both Day 28 and Day 56 in the UO SAF 02 group (p < 0.001), indicating early improvement that was further enhanced with continued treatment. In contrast, the placebo group demonstrated only minimal changes that did not reach statistical significance.

Between-group comparisons demonstrated statistically significant differences at both post-baseline time points (p < 0.001), indicating a clear treatment effect of UO SAF 02 compared with placebo. These differences were evident by Day 28 and became more pronounced at Day 56. At Day 56, the between-group difference in change from baseline was -5.97 (95% CI: -6.64 to -5.30; Cohen’s d = 4.66), indicating a substantial improvement in sleep quality (Table 5).

Serum Cortisol

**Figure 4:**
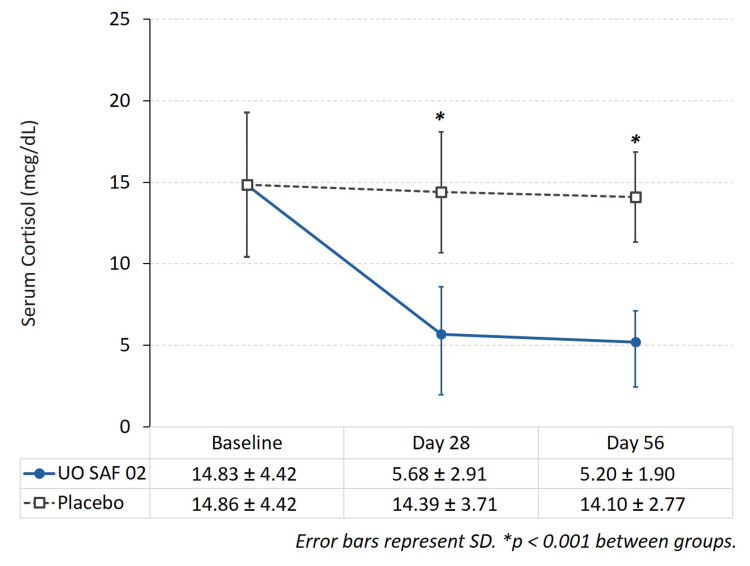
Changes in serum cortisol levels (µg/dL) from baseline to Day 56 (ITT population) ITT: intention to treat

Serum cortisol levels showed a marked and early reduction in the UO SAF 02 group over the study period (Table 6, Figure 4).

**Table 6:** Changes in serum cortisol levels (µg/dL) from baseline to Day 56 (ITT population) Data presented as Mean ± SD. Δ indicates change from baseline. Between-group comparisons were performed using independent sample t-tests. 95% confidence intervals and Cohen's d values were calculated for between-group differences in change scores. ITT: intention to treat

Timepoint	UO SAF 02 (Mean ± SD) (n = 28)	Placebo (Mean ± SD) (n = 28)	Mean difference (95% CI)	p-value	Effect size (Cohen's d)
Baseline	14.83 ± 4.42	14.86 ± 4.42	—	—	—
Day 28	5.68 ± 2.91	14.39 ± 3.71	—	< 0.001	—
Day 56	5.20 ± 1.90	14.10 ± 2.77	—	< 0.001	—
Δ Day 28	-9.15 ± 4.16	-0.48 ± 1.97	-8.67 (-10.41 to -6.93)	< 0.001	2.66
Δ Day 56	-9.63 ± 5.46	-0.76 ± 2.74	-8.87 (-11.28 to -6.46)	< 0.001	2.05

Within-group analysis revealed statistically significant reductions from baseline at both Day 28 and Day 56 in the UO SAF 02 (p < 0.001), suggesting a rapid onset of effect that was sustained throughout the study period. In contrast, cortisol levels in the placebo group remained largely unchanged, with no statistically significant differences observed over time (p > 0.05).

Between-group comparisons showed a statistically significant reduction in serum cortisol levels in the UO SAF 02 group compared with placebo at both post-baseline time points (p < 0.001). These differences were evident as early as Day 28 and became more pronounced at Day 56. At Day 56, the between-group difference in change from baseline was -8.87 (95% CI: -11.28 to -6.46; Cohen’s d = 2.05), further supporting the efficacy of UO SAF 02 in modulating this stress biomarker (Table 6).

Safety and Tolerability

UO SAF 02 was well tolerated, with no serious adverse events reported. A total of four mild adverse events were observed, including three (10.7%) in the UO SAF 02 group and one (3.6%) in the placebo group (Table 7). All events were mild, transient, and resolved without intervention.

**Table 7:** Summary of adverse events

Adverse event	UO SAF 02 (n = 28)	Placebo (n = 28)
Drowsiness	1 (3.6%)	0 (0%)
Nausea	1 (3.6%)	1 (3.6%)
Diarrhea	1 (3.6%)	0 (0%)
Total events	3 (10.7%)	1 (3.6%)

No clinically significant changes were observed in laboratory parameters, vital signs, or physical/systemic examinations, as all observed variations remained within normal reference ranges and were not associated with clinical symptoms or medical intervention.

## Discussion

The present study demonstrated that supplementation with UO SAF 02 significantly improved mood-related outcomes in adults with self-reported low mood and subclinical to mild depressive symptoms. Improvements were observed across multiple domains, including overall mood disturbance, depressive symptoms, affective balance, sleep quality, and serum cortisol levels, compared with placebo. The findings indicated that the intervention exerts beneficial effects across both psychological and physiological domains. The observed trajectory suggests both rapid onset and sustained efficacy, with clinically meaningful improvements evident as early as Day 28 and a further enhanced effect at Day 56. The effect sizes observed in the present study should be interpreted in the context of the relatively low baseline variability and the use of change scores, which may amplify standardized effect estimates, particularly in homogeneous subclinical populations.

The findings of the present study are consistent with a substantial and growing body of evidence supporting the antidepressant effects of saffron. Multiple randomized controlled trials and meta-analyses have demonstrated that saffron supplementation significantly improves depressive symptoms compared with placebo, with large effect sizes reported in meta-analytic evaluations [16,19,32]. Systematic reviews have further confirmed that saffron is significantly more effective than a placebo in improving depressive symptoms and related outcomes [33]. Comparative studies also suggest that saffron exhibits efficacy comparable to conventional antidepressants, including selective serotonin reuptake inhibitors, with fewer adverse effects [16,17,34,35].

Importantly, while the majority of prior studies have focused on individuals with clinically diagnosed depression, the present study extends these findings to individuals with low mood and subclinical to mild depressive symptoms. This population is often underrepresented in clinical research despite experiencing clinically meaningful impairment and an increased risk of progression to major depressive disorder [1,7,36]. Emerging evidence suggests that saffron may improve mood and emotional well-being in non-clinical or subthreshold populations; however, data in this group remain limited [22,37]. The present findings, therefore, contribute important evidence supporting the efficacy of saffron in this underserved population.

In addition to improvements in depressive symptoms, the present study demonstrated significant benefits across multiple psychological domains, including affective balance, stress, and sleep quality. Improvements in positive and negative affect are consistent with previous randomized trials reporting enhanced emotional well-being following saffron supplementation [32]. Furthermore, the observed reductions in overall psychological distress, as reflected by total DASS-21 scores, align with prior evidence supporting the broad-spectrum effects of saffron across interconnected psychological domains [20,38].

The observed improvement in sleep quality is also supported by systematic reviews indicating that saffron supplementation enhances sleep duration and quality [23]. Given the bidirectional relationship between sleep disturbances and depressive symptoms, improvements in sleep may have contributed to the overall benefits in mood and emotional regulation [39,40].

A key finding of the present study is the significant and sustained reduction in serum cortisol levels. The hypothalamic-pituitary-adrenal (HPA) axis regulates the physiological stress response through the sequential release of corticotropin-releasing hormone (CRH), adrenocorticotropic hormone (ACTH), and cortisol. Dysregulation of this pathway is frequently observed in depressive and stress-related disorders and is associated with altered cortisol secretion and impaired stress adaptation [41,42]. Reduced HPA axis hyperactivity and normalization of cortisol levels have been associated with improved mood regulation and stress resilience [41,43,44].

Saffron has also been reported to influence neuroendocrine regulation, including modulation of the HPA axis and reduction of cortisol levels [33,39]. The reduction in serum cortisol observed in the present study may reflect improved regulation of stress-response pathways. Furthermore, emerging evidence suggests that saffron may influence neurotrophic signaling pathways, including those involving brain-derived neurotrophic factor, thereby supporting neuronal plasticity and resilience [33].

The observed clinical benefits may be explained by the multifaceted neuropsychotropic properties of saffron and its bioactive constituents, including crocin and safranal [24,45,46]. Crocin is a water-soluble carotenoid. Following oral administration, it is extensively converted to crocetin, which is absorbed systemically and is believed to contribute to the biological effects of saffron [47,48]. While safranal is a lipophilic, volatile monoterpene that is rapidly absorbed following oral administration, it undergoes extensive metabolism, resulting in relatively low systemic exposure and a short plasma half-life, which may influence its bioavailability [48,49]. These constituents exert distinct but complementary pharmacological effects. Crocin has been associated with antioxidant, anti-inflammatory, neuroprotective, and monoaminergic effects [18,46,50], while safranal has been linked to modulation of serotonergic neurotransmission as well as anxiolytic and antidepressant activities [51,52]. Together, these complementary mechanisms may contribute to the mood-enhancing effects of saffron [11,40,53].

The pharmacokinetics and bioavailability of saffron bioactive compounds are important determinants of its clinical efficacy. Crocin, a major active constituent, exhibits limited stability and is rapidly hydrolyzed to crocetin in the gastrointestinal tract prior to absorption [54]. The bioavailability of saffron constituents is further influenced by gastrointestinal conditions, solubility, and formulation characteristics, highlighting the importance of delivery systems in optimizing therapeutic outcomes [55,56]. Standardization of key bioactive components of saffron extracts has been shown to enhance pharmacological activity and improve reproducibility of clinical outcomes [57]. In this context, the extent of effect observed in the present study may also be partially influenced by the formulation approach of UO SAF 02, which is designed to enhance stability and bioavailability. The solid-liquid fusion technology approach to formulate UO SAF 02 involves incorporating saffron-derived actives into the matrix to create a structured delivery system. The matrix is intended to protect sensitive constituents from degradation in the gastric environment and enable their gradual release in the intestinal phase, thereby facilitating improved absorption and utilization. However, because pharmacokinetic parameters were not assessed in this study, this hypothesis remains speculative and warrants further mechanistic investigation.

The safety findings of the present study are consistent with previous clinical studies demonstrating that saffron is generally well tolerated at therapeutic doses. Only four mild adverse events were reported across both study groups, and no serious adverse events or discontinuations due to adverse events occurred. Reported adverse events, including drowsiness, nausea, and gastrointestinal discomfort, are similar to those observed in earlier saffron trials and systematic reviews, where adverse events were typically mild, transient, and comparable to placebo [32,58,59]. Importantly, no clinically significant safety concerns were identified during the study, supporting the favorable tolerability profile of UO SAF 02.

Previous clinical studies have generally evaluated saffron at doses of 20-30 mg/day for mood-related outcomes, with a favorable safety profile and adverse event rates comparable to placebo [53]. Doses substantially higher than those used therapeutically may be associated with toxicity, with reports suggesting that doses above 5 g may produce toxic effects, whereas doses above 10 g have been associated with abortifacient effects, and very high doses may be life-threatening [60,61].

Saffron is generally well tolerated; however, caution may be warranted in individuals with bleeding disorders or those receiving antihypertensive or anticoagulant medications because of potential pharmacological interactions [62,63]. While saffron has been studied primarily in adult populations, evidence in pediatric, elderly, pregnant, and lactating populations remains limited. Therefore, use during pregnancy, particularly at high doses, should be approached with caution because of reported uterine stimulant effects [53,61].

Overall, these findings are clinically relevant for individuals with low mood and subclinical to mild depressive symptoms. The demonstrated efficacy, early onset, and favorable tolerability of UO SAF 02 suggest that this saffron-based intervention may provide a safe and accessible approach to support mood regulation in this population. Early intervention at this stage may help prevent progression to more severe depressive disorders. The observed improvements across multiple domains, including mood, affect, sleep, and stress biomarkers, further indicate broad benefits extending beyond symptom reduction, contributing to overall psychological well-being.

Strengths and limitations

The strengths of the present study include its randomized, double-blind, placebo-controlled design, which minimizes bias and enhances internal validity. The use of multiple validated outcome measures enabled a comprehensive assessment of both psychological and physiological parameters. Additionally, the inclusion of individuals with low mood and subclinical to mild depressive symptoms addresses an important and under-researched population. However, certain limitations should be acknowledged. The sample size was relatively modest, and larger studies are required to confirm these findings. The study duration was limited to 56 days, and longer-term studies are needed to assess the sustainability of effects. Although validated instruments were used, reliance on self-reported measures may introduce subjective bias; however, the inclusion of objective biomarkers such as cortisol strengthens the robustness of the findings. Finally, generalizability may be limited to similar populations and may not extend to individuals with clinically diagnosed depression or more severe psychiatric conditions.

Future studies should include larger, multi-center trials to validate the findings of this study. Longitudinal research is needed to assess the durability of effects and to evaluate whether such interventions may influence the progression to clinically significant depressive disorders. Further mechanistic and pharmacokinetic studies would also help to better understand the pathways underlying the observed effects.

## Conclusions

In this randomized, double-blind, placebo-controlled trial, UO SAF 02 significantly improved mood-related outcomes in adults with low mood and subclinical to mild depressive symptoms compared with placebo. Improvements were observed across multiple domains, including mood disturbance, affect, sleep quality, and serum cortisol levels, with effects evident by Day 28 and sustained through Day 56. These findings support the potential of UO SAF 02 as a well-tolerated intervention for mood regulation in subclinical populations. However, larger and longer-term studies are required to confirm these results and to further evaluate its role in the prevention of clinically significant depressive disorders.
